# Non-Cardiac Amyloidosis Findings Are Not Increased in African American Carriers of *TTR* V142I with Heart Failure and/or Arrhythmia

**DOI:** 10.3390/jpm14030271

**Published:** 2024-02-29

**Authors:** Scott Kaniper, Dorret Lynch, Samuel M. Owens, Larisa Ibric, Yuliya Vabishchevich, Nana Nyantakyi, Fan Chun, Lionel Sam, Carly Fabrizio, Eman Hamad, Glenn S. Gerhard

**Affiliations:** 1 Lewis Katz School of Medicine, Temple University, Philadelphia, PA 19140, USA; 2 Department of Medicine, Section of Cardiology, Temple University Health System, Philadelphia, PA 19140, USA

**Keywords:** transthyretin, amyloid, cardiomyopathy, hereditary ATTR-CM, African American ancestry

## Abstract

Transthyretin amyloid cardiomyopathy (ATTR-CM) is a progressive systemic disease involving the extracellular deposition of misfolded transthyretin protein. The hereditary subtype is caused by mutations in the transthyretin (*TTR)* gene. An estimated 2–3% of individuals of African American (AA) ancestry carry the p.Val142Ile (V142I, also referred to as V122I) *TTR* pathogenic variant. The non-specific clinical nature of ATTR-CM makes it challenging to diagnose clinically, and the high allele frequency of *TTR* V142I suggests that many patients with hereditary ATTR-CM may not have been tested. An analysis of electronic health record data from over 13,000 AA patients with a diagnostic code for heart disease or arrhythmia who also had additional amyloid-related findings were not diagnosed with amyloidosis at higher rates than those with heart failure or arrhythmia who did not have additional amyloid-related clinical diagnoses. Similarly, after genotyping 666 AA patients with heart failure or arrhythmia, *TTR* V142I carriers appeared to be clinically indistinguishable based on amyloid-related non-cardiac diagnoses from those who did not carry the allele. No additional *TTR* gene sequence variants were found in the *TTR* wildtype V142V patients with heart failure or arrhythmia who had additional amyloid-related diagnoses. Genetic testing for ATTR-CM may be important for timely diagnosis.

## 1. Introduction

Transthyretin amyloid cardiomyopathy (ATTR-CM) is a progressive systemic disease involving the extracellular deposition of misfolded transthyretin protein [1]. Transthyretin is produced in the liver and circulates in the blood as a tetramer transporting thyroxin and retinol [2]. There are both mutation-related (hereditary) and wildtype (previously referred to as senile cardiac amyloidosis) forms of ATTR-CM [3]. Mutations in the transthyretin gene (*TTR)* increase tetramer dissociation, misfolding, and the aggregation of monomers into oligomeric amyloid fibrils, which can deposit in the heart and peripheral nervous system. The kinetics of monomer oligomerization appear to be related to amyloidogenicity, with disease-causing variants increasing the rates of aggregation. Increasing age is also associated with *TTR* tetramer instability.

The hereditary subtype is caused by autosomal dominantly acting mutations in the *TTR* [4]. More than 100 likely/pathogenic *TTR* variants have been described [5], with most mutations classified as single-nucleotide variants that cause substitutions of amino acids in the *TTR* protein monomer. There is extreme variability in the penetrance, expressivity, age of onset, and speed of progression, and whether neuropathy, cardiomyopathy, or other symptoms are the predominant phenotype is mutation-dependent. For example, the V30M variant can occur as an early-onset (<50 years of age) neurological disorder, which is found more frequently in Japan, Portugal, and Sweden than the later-onset disease, which also has cardiac manifestations. The T60A *TTR* variant that appears to have originated in Ireland also has both neurological- and cardiomyopathy-related symptoms. The allele frequencies of these variants are rare compared to p.Val142Ile (V142I, also referred to as V122I), carried by an estimated >3% of individuals of African American (AA) ancestry. V142I has been associated primarily but not exclusively with ATTR-CM [6]. A systematic literature review of the prevalence and outcomes of V142I ATTR-CM based on an aggregate sample size of ~150,000 found that the adjusted odds ratio for heart failure (HF) ranges from ~1.5 to 1.8 [7].

Despite the high prevalence of the *TTR* V142I variant in AA, there appears to be a substantial divergence between the number of *TTR* V142I carriers and diagnosed cases of ATTR-CM [8], suggesting that many patients, perhaps most, are undiagnosed, particularly those with heart failure who do not have overt clinical findings to suggest amyloidosis. In addition, ATTR-CM-related ventricular hypertrophy or heart failure may be difficult to distinguish from other prevalent causes of heart disease, such as hypertensive and hypertrophic cardiomyopathy, which are also highly prevalent [6]. Similarly, several types of arrhythmias, particularly atrial fibrillation, can occur with ATTR-CM [9] but are often ascribed to other causes. Some studies have reported non-cardiac manifestations of amyloidosis in *TTR* V142I carriers, including polyneuropathy and carpal tunnel syndrome, though the actual sample sizes of older patients were small [10]. The biological, genetic, and environmental factors that lead to the phenotypic penetrance and variable expressivity of ATTR-CM, thus, remain largely unknown.

Therapies have been developed to treat ATTR-CM, including antisense oligonucleotides, small interfering RNAs (ribonucleic acids), and CRISPR–Cas9 (clustered regularly interspaced short palindromic repeats-CRISPR-associated protein 9) gene editing, as well as small-molecule drugs [11]. Decreasing *TTR* protein translation in the liver to decrease circulating blood levels can produce corresponding reductions in amyloid deposition. Two primary approaches that have been developed and are now approved for clinical use are antisense oligonucleotides (ASOs) and small interfering RNAs (siRNAs). sASOs are short (~20 bases) single-strand oligonucleotides that are synthesized to hybridize to complementary sequences in the *TTR* mRNA. The ASO DNA-*TTR* mRNA hybrid is then targeted by the cell for degradation by ribonuclease RNase H1, depleting mRNA levels and reducing translation of the monomer protein. Similarly, siRNAs are slightly longer double-strand synthetic RNAs that also hybridize to the *TTR* mRNA, which is then degraded by the RNA-induced silencing complex (RISC). Although drugs from both of these classes have been approved for *TTR*-related polyneuropathy, siRNA has been associated with effectiveness against ATTR-CM in patients with mixed disease. The use of CRISPR–Cas9 to inactivate *TTR* in vivo is also being investigated. Small-molecule drugs to prevent TTR tetramers from dissociating have also been approved.

The non-specific nature of ATTR-CM makes it challenging to diagnose clinically, and the high allele frequency of *TTR* V142I suggests that many patients with hereditary ATTR-CM have not been thoroughly or formally evaluated and tested. With the growing repertoire of recently developed drugs to treat *TTR*-related amyloidosis [12], a significant health disparity exists for undiagnosed patients. We sought to determine whether carriers of *TTR* V142I of AA ancestry with heart failure or arrhythmia had extra-cardiac manifestations of amyloidosis, which could help suggest the diagnosis of amyloidosis. We studied patients seen in the Temple University Hospital System (TUHS), a catchment area with a high proportion of residents who self-identify as AA [13] and a high rate of cardiovascular disease. We also determined whether non-carriers of *TTR* V142I with heart failure or arrhythmia plus other non-cardiac manifestations of amyloidosis had other *TTR* variants.

## 2. Materials and Methods

### 2.1. Participants

Samples for DNA (deoxyribonucleic acid) isolation were obtained from clinically ordered EDTA anti-coagulated whole blood samples analyzed by the Temple University Health System (TUHS) Clinical Laboratory. The samples were collected from May 2021 to 2022 and stored for up to three days prior to biobanking. The Institutional Review Board (IRB) of Temple University approved the research. The electronic health record data of patients treated at Temple University Hospital, an urban academic medical center in Philadelphia, Pennsylvania, USA, were retrospectively analyzed using ICD-10 (International Classification of Diseases, Tenth Revision) codes (Table 1).

### 2.2. Blood Samples and DNA Preparation

Genomic DNA for sequencing was isolated using the EZ1 DNA Blood 200 μL kit on the EZ1 Advanced XL instrument (Qiagen, Valencia, CA, USA). The DNA was extracted from EDTA anti-coagulated whole blood using the Qiagen MagAttract DNA Blood Midi M48 kit and the Qiagen BioRobot M48 Workstation (Qiagen) according to the manufacturer’s directions. Quantification of the extracted DNA was performed using the NanoDrop ND-1000 spectrophotometer (NanoDrop Technologies, Wilmington, DE, USA).

### 2.3. TTR V142I Genotype Analysis

Genotyping for the p.Val142Ile (chr18:31598655, c.424G>A, rs76992529) variant was performed using a custom TaqMan genotyping assay that used probe fluorophores, VIC and FAM, to distinguish G from A on the Applied Biosystems QuantStudio 7 Flex System (Thermo Fisher Scientific Waltham, MA USA). The primer sequences were the forward primer CTGAGCCCCTACTCCTATTCCA, reverse primer GGAGGAGAAGTCCCTCATTCCTT, Probe 1 (VIC) ACGGCTGTCGTCACCAA, and Probe 2 (FAM) ACGGCTGTCATCACCAA, with the positions of the forward and reverse primers in the TTR gene sequence, as shown in Method S1. The DNA was genotyped according to the manufacturer’s protocol. The reaction components for each genotyping reaction were as follows: 10 ng of DNA, 5 μL of TaqMan Genotyping Master Mix (Applied Biosystems, Foster City, CA, USA), 0.25 μL of assay mix (40×), and water up to a total volume of 10 μL. The cycling conditions were 1 cycle at 60 °C for 30 s, followed by 1 cycle at 95 °C for 10 min, followed by 40 cycles of 95 °C for 15 s and 60 °C for 1 min, followed by 1 cycle at 60 °C for 30 s. The reaction was then analyzed by TaqMan Genotyper Software version 1.6.0.

### 2.4. TTR Gene Sequencing

The entire *TTR* gene was amplified on the Veriti Thermocycler as a 7792 bp fragment using long PCR (polymerase chain reaction) with the long PCR forward primer ACGAATGTTCCGATGCTCTAAT and reverse primer TGAGTTGCTGCAGGTGTATC. Following gel purification, the extracted DNA was verified by gel electrophoresis (Method S2), excised and purified, quantified, and then subjected to Sanger sequencing (Genewiz, Inc., South Plainfield, NJ USA) using 10 μL of purified PCR product, 2.5 μL of 10 μM sequencing primer, and 2.5 μL H_2_O. The sequencing primers (diagrammed in Method S2) were exon 1 forward primer ACGAATGTTCCGATGCTCTAAT and reverse primer AGTTCAAGTCCCAGCTCAGTAAG, exon 2 forward primer TGGGATCAGTGTGTAATTCTTGTTT and reverse primer CACAGCTAGAGGAGAGGAGTTCT, exon 3 forward primer AGGAGTTTTCCCTACTTCTGACTTA and reverse primer ATAGGAAAGGGAACCTTTGGTCATT, and exon 4 forward primer ACTTCCGGTGGTCAGTCATGTG and reverse primer TTAATACGTGCTTTGCTTGCAAGA. To identify the variants, FASTA sequence data were aligned to the reference genome using BLAST, and the fluorescent trace electrophoretograms were visually inspected.

### 2.5. Bioinformatics Analysis

Because the automated base calling of sequence data using the ABI 3730xl DNA Analyzer Sequencing Analysis KB Basecaller software cannot distinguish coincident base peaks characteristics of heterozygous variants, each Sanger sequencing capillary electrophoretogram was visually inspected to identify and confirm the variants. The NCBI BLAST tool (https://blast.ncbi.nlm.nih.gov/Blast.cgi, accessed on 8 January 2024) was used to align the DNA sequence to the *TTR* sequence (NG_016441.1) and to translate the DNA sequence to its corresponding protein sequence. The NCBI gene database (https://www.ncbi.nlm.nih.gov/gene, accessed on 8 January 2024) was used to access *TTR* orthologs.

### 2.6. Statistical Analysis

All statistical tests were two-sided and conducted using Prizm 10.0, with *p* ≤ 0.05 considered statistically significant.

## 3. Results

Electronic health record data were extracted for over 13,000 TUHS AA patients with a diagnostic code for heart failure or arrhythmia as well as codes for other manifestations of ATTR-related amyloidosis, as described by expert consensus recommendations [14]. The ICD-10 codes used for data extraction are shown in Table 1.

Of the 13,029 AA patients with a diagnosis of either heart failure or arrhythmia, only 82 (0.63%) also had a diagnostic code for amyloidosis due to any cause (Table 2). Of the various amyloidosis diagnoses (Table S1), over half were coded as either “Organ-limited amyloidosis”, “Amyloidosis, unspecified”, or “Other amyloidosis”. The percentages of AA patients with a diagnosis of heart failure or arrhythmia and amyloidosis who also had non-cardiac amyloidosis findings varied by diagnosis. The percentages of patients with heart failure or arrhythmia and amyloidosis and no other diagnoses were not different from those who also had amyloidosis-related diagnoses (including spinal stenosis, spinal radiculopathy, bilateral carpal tunnel, orthostatic hypotension, bladder dysfunction, multi/chronic diarrhea, numbness or tingling, eye disease, spinal stenosis or spinal radiculopathy and eye diseases, multi/chronic diarrhea and spinal stenosis, multi/chronic diarrhea, and spinal radiculopathy). Only the percentage of patients with heart failure or arrhythmia and gastroparesis was nominally different (*p* = 0.029, Fisher’s exact test). However, this result was not significant after correction for multiple testing. In contrast, the percentage of patients with heart failure or arrhythmia and amyloidosis and left ventricular hypertrophy was over four-fold higher (nominal *p* = 0.015, Fisher’s exact test) than that of those with heart failure or arrhythmia and amyloidosis without left ventricular hypertrophy. Similarly, the percentage of patients with heart failure or arrhythmia and amyloidosis was over eight-fold higher in those who also had an atrioventricular block (*p* = 0.022, Fisher’s exact test).

**Figure 1:**
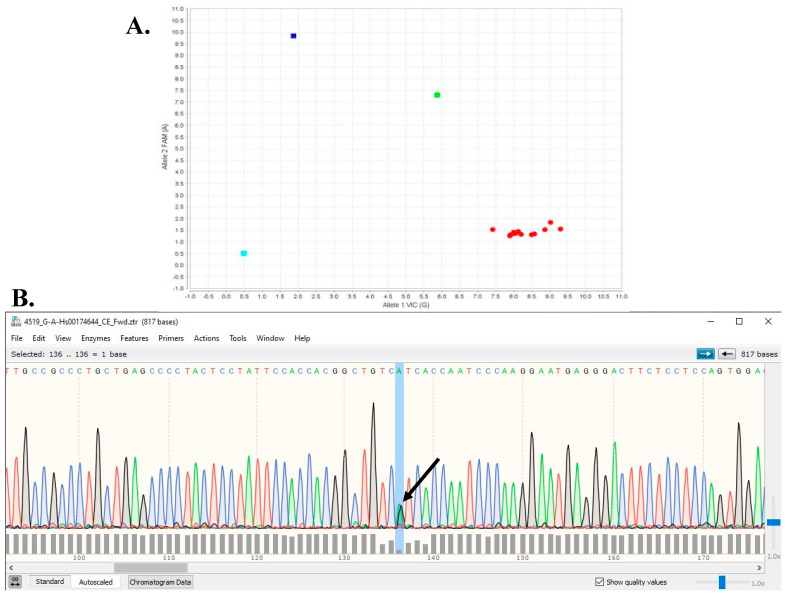
(**A**) Example of *TTR* Taqman genotyping assay result for *TTR* V142I. Blue square is homozygous V142I/V142I, green square is heterozygous V142V/V142I, and red squares are homozygous reference V142V/V142V (turquoise square is baseline negative control for assay). (**B**) Example of Sanger sequencing chromatogram. Each fluorescence tracing peak color corresponds to each of the four DNA bases, i.e., green = A, red = T, black = G, and blue = C peaks Arrow indicates overlapping shorter A (green) and G (black) peaks for heterozygous V142V/V142I.

**Figure 2:**
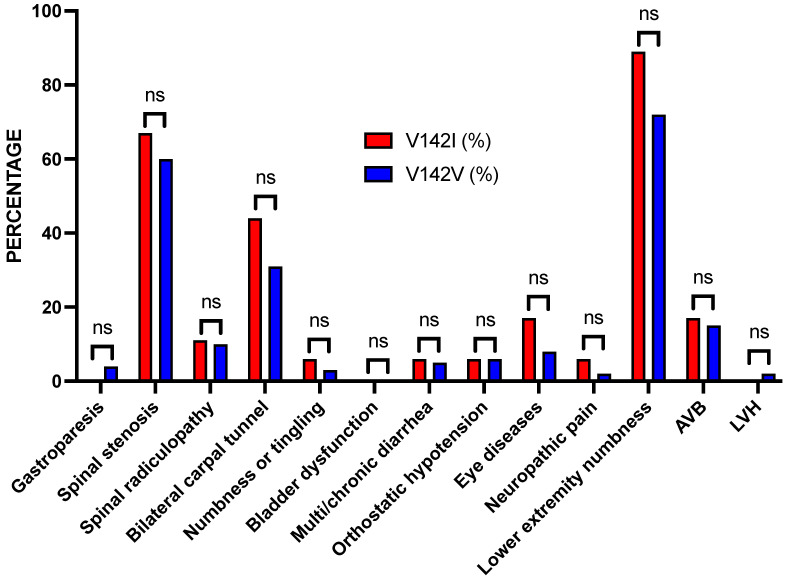
Bar graph of the percentages of patients with the *TTR* V142I variant or the reference V142V sequence by diagnostic category. Although the percentages of most of the non-cardiac findings were higher in the *TTR* V142I carriers, all were not significant (ns).

To estimate the prevalence of *TTR* V142I, the DNA obtained from the blood samples of 666 TUHS AA patients with a diagnosis of heart failure or arrhythmia included in a biobank of samples was genotyped. The TaqMan genotyping (Figure 1A) assay was an efficient and robust method to identify V142I. Approximately 2.7% of the patients carried a *TTR* V142I allele (18/666; 1 homozygote, 17 heterozygotes). The *TTR* V142I homozygote was >65 years and female, with a diagnosis of HF or arrhythmia plus spinal stenosis, bilateral carpal tunnel syndrome, multi/chronic diarrhea, eye disease, and lower extremity numbness. Of the 18 *TTR* V142I allele carriers, 38.9% were male, and 50% were 65 years of age or older. This percentage of males was not different from those <65 years of age (*p* < 0.475, Fisher’s exact test). Similar percentages for age and sex distribution were found for the V142V reference sequence individuals. Although the percentages of most of the non-cardiac findings were higher in the *TTR* V142I carriers, none were statistically significant (Table 3 and Figure 2).

We then sequenced (Figure 1B) the 27 patients with a diagnosis of heart failure or arrhythmia who did not carry *TTR* V142I, i.e., they had the *TTR* V142V wildtype but also had other amyloidosis-related diagnoses (Table S1). All 27 had bilateral carpal tunnel, 25 had lower extremities numbness, 23 had spinal radiculopathy, and 21 had spinal stenosis. The number of amyloidogenic diagnoses ranged from 2 to 8, with a median of 5. Variants were identified in 23/27 patients (Table S2). A total of 22/27 patients had intronic variants, 3 patients had variants in the 3′ untranslated coding region, and no missense or other exon variants were found in any of the patients. Accessing the gnomAD (genome Aggregation Database) database [15] revealed that of the 23 TTR missense variants, only 4 other than V142I were present in the African American population: p.Ile88Leu, p.Thr80Ile, p.Val50Met, and p.Thr126Asn (Table S3). The allele frequency in AA for these four were 4/74,294, 1/59,116, 1/75,034, and 1/41,466, respectively. In contrast, in the European non-Finnish population, the respective allele frequencies for the four variants were 17/1,179,982, 0/418,132, 62/1,179,988, and 0/68,038. Although there is no information on any clinical conditions in individuals in gnomAD, given the later age of onset and variable penetrance and expressivity of ATTR-CM, these data suggest that apart from V142I, *TTR* variants are rare in AA. Valine at position 142 is also highly conserved, present in about 90% of species in the NCBI (National Center for Biotechnology Information) Gene database, with *TTR* orthologs.

## 4. Discussion

The data from the TUHS indicate that approximately 0.63% of AA patients with a diagnosis of either heart failure or arrhythmia also carry a diagnosis of amyloidosis. In patients with either heart failure or arrhythmia, plus the diagnosis of gastroparesis, bilateral carpal tunnel, orthostatic hypotension, or chronic diarrhea, the percentage of patients with a diagnosis of amyloidosis increased by 2–4-fold, though none of the increases met statistical significance after correcting for multiple testing. Similarly, after genotyping 666 AA patients with a diagnosis of either heart failure or arrhythmia, the percentages of V142I and V142V patients did not have any significant differences in the diagnoses of 11 non-cardiac amyloid-related diagnoses and 2 cardiac diagnoses. These data further suggest that *TTR* V142I in AA predominantly manifests as a cardiac phenotype related to heart failure [16,17,18], supported by a large systematic review of the literature [7]. The data showing the contrary have been largely based on very small sample sizes [19].

We found no other *TTR* variants in AA patients with either heart failure or arrhythmia who also had multiple other diagnoses associated with *TTR* amyloidosis. Given the relatively cardiac-specific phenotype of TTR V142I ATTR-CM, analyzing a small sample size of heart failure patients with additional amyloid diagnoses likely did not enrich for patients with *TTR* mutations. In addition, based on the gnomAD database, the frequency of non-V142I *TTR* mutations is very low.

Why V142I has a high allele frequency for a dominantly acting pathogenic variant is not clear. Humans who lack *TTR* do not appear to have been described [20]; thus, the gene likely has some essential function. However, mice without *TTR*, i.e., *TTR* knockouts, appeared to be phenotypically unaffected, with normal viability and fertility [21]. The initial designation of TTR as prealbumin, because its migration was ahead of albumin in serum protein electrophoresis, was changed to thyroxine-binding prealbumin following the discovery that it transported thyroid hormone [22]. After it was also found to bind to retinol-binding protein, transthyretin was found to transport (trans-) thyroid hormone (-thyr-) and retinol-binding protein (-retin). The valine at position 142 is highly conserved, suggesting that this particular amino acid is important for *TTR* function. The substitution of isoleucine for valine appears to destabilize *TTR* tetramers [23]. The 142 valine position appears to be in a conserved β-sheet in strand H of the *TTR* protein, which corresponds to a serine in a highly conserved YRGS motif that is present in non-vertebrate *TTR* homologs but absent from vertebrates [24]. The function of this YRGS motif is not known.

How the V142I variant predisposes someone to cardiac manifestations in favor of neurological ones is not clear. The V142I variant appears to destabilize the *TTR* homotetramer relative to the reference V142V homotetramer [25]. Amino acid 142 is on the periphery of the H strand β-sheet, which forms part of the quaternary structural interface of the homotetramer. Antiparallel β-sheet interactions occur between the H strands of two monomers, contributing to the stability of the dimer interface. The side chain of the mutant 142 isoleucine has altered interactions (relative to the valine at 142) with the side chains of the amino acids of the neighboring subunit. The altered interactions at the dimer–dimer interface cause the tetramer instability. The V142I *TTR* monomer that results can undergo a rapid partial denaturation, which allows for self-assembly into amyloid fibrils. The tissue specificity of the amyloid deposition may be related to the physico-chemical characteristics of the resulting fibrils [26].

With the relatively high allele frequency of V142I and the estimated adjusted odds ratio for the risk of heart failure estimated at ~1.5–1.8 in *TTR* V142I carriers [7], many patients are either undiagnosed or at risk for being diagnosed in the future. In a study of 278 AA individuals who were ≥60 years old with heart failure, 19 (6.8%) had positive cardiac technetium-99m-pyrophosphate imaging indicative of amyloid, with 7 (37%) of these individuals genotyping as carriers of V142I [8]. These data further support the likely underdiagnosis of ATTR-CM due to *TTR* V142I.

Currently, the main diagnostic algorithm for patients with suspected cardiac amyloidosis utilizes genetic testing as a near-final step. Diagnostic modalities recommended prior to genetic analysis include electrocardiography, echocardiography, cardiac magnetic resonance imaging, biomarkers, serum kappa/lambda free light chain ratio, serum and urine immunofixation, and endomyocardial biopsy and/or myocardial scintigraphy with bone avid tracers [27,28]. Although these methods can detect manifestations of ATTR-CM, the wide array of clinical signs and symptoms and heterogeneity of the cardiac phenotype can result in diagnostic delays or misdiagnosis [29]. Genetic testing as an almost final diagnostic step may also be overlooked, compounded by the prevailing view that genetic testing is expensive. However, genetic testing has now become low-cost (and for TTR amyloidosis can be free), non-invasive, and is now widely used in medicine [30]. The use of genetic testing would both streamline the diagnostic process and improve the rates of accurate diagnosis. This may be particularly important for V142I ATTR-CM, which may manifest a more aggressive cardiac phenotype and poorer prognosis [31] and an increase in all-cause mortality relative to V142I noncarriers [17].

Several *TTR*-directed treatments are now available for ATTR-CM. However, symptomatic therapy with heart failure medications is commonly used, particularly prior to the diagnosis. The clinical benefit of guideline-directed therapy for patients with HFrEF (heart failure with reduced ejection fraction) in ATTR-CM, including beta-blockers and angiotensin-converting enzyme inhibitors, is not clear [32]. Other drugs, such as calcium channel blockers and digitalis, may be contraindicated [33]. In addition, delayed diagnosis will result in the delayed initiation of appropriate treatment. ATTR-CM patients who experienced delays in diagnosis and treatment were found to have poor prognostic indicators, including a higher NYHA (New York Heart Association) classification, increased cardiac biomarkers, and lower health-related quality of life [34]. For this reason, using genetic testing to diagnose ATTR-CM in heart failure or arrhythmia in AA patients with or without additional clinical indications may be important for the timely initiation of appropriate therapy.

## Figures and Tables

**Table 1:** ICD-10 codes and corresponding diagnoses used in analysis of electronic health record data to identify patients with manifestations of ATTR-related amyloidosis.

Heart Disease	
I11.0	Hypertensive heart disease with heart failure
I13.0	Hypertensive heart disease and chronic kidney disease with heart failure
I49	Other cardiac arrhythmias
I50	Heart failure
NA *	Family history of heart failure
Autonomic Dysfunction	
I95.1	Orthostatic hypotension
N31.8	Other neuromuscular dysfunction of bladder
N31.9	Neuromuscular dysfunction of bladder, unspecified
Gastrointestinal Autonomic Disease	
K31.84	Gastroparesis
NA *	Chronic diarrhea
NA *	Multiple episodes of diarrhea (patients with >3 encounters)
Sensory and/or Motor Neuropathy	
NA *	Bilateral numbness or tingling in extremities
Other Neurological Effects	
G56.03, Z.98.890	Bilateral carpal tunnel
M54.1	Spinal radiculopathy
M48	Spinal stenosis
Eye Disease	
NA *	Blindness, retinal detachment, or vitreous opacities
Amyloid	
E85	Amyloidosis
Left Ventricular Hypertrophy (Lvh)	
NA *	LVH due to hypertensive disease, with heart failure
NA *	LVR due to hypertensive disease, without heart failure
NA*	LVH
Atrioventricular Block	
I44	Atrioventricular block

* NA—searched diagnosis name.

**Table 2:** Non-cardiac amyloidosis diagnoses in TUHS AA patients with heart failure (HF) or arrhythmia.

Diagnosis	Count	>65 Years	Male	HF	Arrhythmia	Plus Amyloid	% Amyloid
HF or Arrhythmia	13,029	59%	46%	10,668	3567	82	0.63
HF or Arrhythmia and Spinal Stenosis	3502	58%	37%	2860	1054	23	0.66
HF or Arrhythmia and Spinal Radiculopathy	980	55%	35%	777	331	6	0.61
HF or Arrhythmia and Bilateral Carpal Tunnel	155	61%	18%	133	43	2	1.29
HF or Arrhythmia and Orthostatic Hypotension	310	89%	54%	263	108	5	1.61
HF or Arrhythmia and Bladder Dysfunction	214	75%	53%	178	65	1	0.47
HF or Arrhythmia and Multi/Chronic Diarrhea	225	62%	31%	195	67	3	1.33
HF or Arrhythmia and Gastroparesis	179	43%	34%	161	33	4	2.23
HF or Arrhythmia and Numbness or Tingling	116	59%	28%	89	41	0	0.00
HF or Arrhythmia and Eye Disease	408	63%	42%	375	89	4	0.98
HF or Arrhythmia and Spinal Stenosis or Spinal Rad. and Eye Diseases	181	61%	34%	162	50	0	0.00
HF or Arrhythmia, Multi/Chronic Diarrhea and Spinal Stenosis	120	68%	26%	102	43	2	1.67
HF or Arrhythmia, Multi/Chronic Diarrhea and Spinal Radiculopathy	39	62%	10%	30	15	0	0.00
HF or Arrhythmia and LVH	252	65%	54%	189	89	7	2.78
HF or Arrhythmia and AVB	1040	75%	51%	859	432	11	1.06
HF or Arrhythmia and LVH and AVB	36	72%	64%	22	22	2	5.56

**Table 3:** Percentages of patients with or without the p.Val142Ile variant by diagnostic category.

Diagnosis	p.Val142Ile (%)	p.Val142Val (%)
Gastroparesis	0	4
Spinal stenosis	67	60
Spinal radiculopathy	11	10
Bilateral carpal tunnel	44	31
Numbness or tingling	6	3
Bladder dysfunction	0	0
Multi/chronic diarrhea	6	5
Orthostatic hypotension	6	6
Eye diseases	17	8
Neuropathic pain	6	2
Lower extremity numbness	89	72
AVB	17	15
LVH	0	2

## Data Availability

All data from this study are presented in the main text or Supplemental Materials.

## Supplementary Materials

The following supporting information can be downloaded at: https://www.mdpi.com/article/10.3390/jpm14030271/s1, Table S1. Non-cardiac clinical manifestations; Table S2. Sanger sequencing variants; Table S3. gnomAD TTR variants; Table S4. AA missense *TTR* variants in gnomAD (v4.0.0) by population; Method S1. Genotyping; Method S2. Sanger sequencing.
