# Supplementary material for: Non-Cardiac Amyloidosis Findings Are Not Increased in African American Carriers of TTR V142I with Heart Failure and/or Arrhythmia
Source: J Pers Med. 2024 Feb 29;14(3):271. doi: 10.3390/jpm14030271 (PMC10971554; doi:10.3390/jpm14030271)
Supplement: Supplementary file 1 [file jpm-14-00271-s001.zip › jpm-2855825-supplementary.pdf]

Supplementary Materials

Non-cardiac amyloidosis findings are not increased in African American carriers of *TTR* V142I with heart failure and/or arrythmia.

Scott Kaniper[1], Dorret Lynch[1], Sam Owens[1], Larisa Ibric[1], Yuliya Vabishchevich[1], Nana Nyantakyi[1], Fan Chun[1], Eman Hamad[1,2], Lionel Sam[1], Carly Fabrizio[1,2], and Glenn S. Gerhard[1]

[1] Lewis Katz School of Medicine at Temple University, Philadelphia, PA 19140, USA
[2] Temple University Health System, Philadelphia, PA 19140, USA

Table S1. Amyloidosis diagnoses

Table S2. Non-cardiac clinical manifestations.

Table S3. Sanger sequencing variants.

Table S4. gnomAD TTR variants.

Method S1. Genotyping

Method S2. Sanger Sequencing.

Table S1. Amyloidosis diagnoses

**Diagnosis**
AL amyloidosis
Amyloidosis
Amyloidosis of skin
Light chain (AL) amyloidosis
Organ-limited amyloidosis
Other amyloidosis
Amyloidosis, unspecified amyloidosis
Amyloidosis, unspecified type
Cardiac amyloidosis
Cerebral amyloid angiopathy
Amyloidosis cutis
Amyloid heart muscle disease
Amyloid heart disease
Amyloid disease
Amyloidosis, unspecified
Primary amyloidosis of light chain type
Secondary systemic amyloidosis
Myeloma associated amyloidosis
Wild-type transthyretin-related (ATTR) amyloidosis
Renal amyloidosis
Pulmonary nodular amyloidosis
Amyloidogenic transthyretin amyloidosis
Secondary amyloidosis
Familial amyloidosis
Idiopathic amyloidosis
Amyloid kidney
Hereditary cardiac amyloidosis
Hepatic amyloidosis
Localized amyloidosis
Lichen amyloidosus
Amyloidosis of lung
Arthropathy in amyloidosis
Dilated cardiomyopathy secondary to amyloidosis
Lichen amyloidosis
Familial amyloid polyneuropathies

Amyloidosis of bladder
Cutaneous amyloidosis
Nephrotic syndrome associated with amyloidosis
Primary amyloidosis of kidney
HCHWA - hereditary cerebral hemorrhage with amyloidosis
Non-neuropathic heredofamilial amyloidosis
Senile cardiac amyloidosis
Amyloid nephropathy
Amyloidosis of dermis
Systemic amyloidosis
Pulmonary amyloidosis
Hereditary cerebral angiopathic amyloidosis
Finnish-type familial amyloidosis (FAP-IV)
Restrictive cardiomyopathy secondary to amyloidosis
Primary amyloidosis
Neuropathic heredofamilial amyloidosis
Nephrotic syndrome due to amyloidosis
Beta-2-microglobulin amyloidosis
Familial amyloid heart disease
Senile amyloidosis
Amyloidosis inherited systemic
Familial transthyretin amyloid cardiomyopathy
Familial amyloid polyneuropathy, 84 ser-for-ile
Primary localized cutaneous nodular amyloidosis
AA amyloidosis
Heredofamilial amyloidosis
Familial renal amyloidosis
AL amyloid nephropathy
Amyloidosis, hereditary

Table S2. Patients of African American ancestry with a diagnosis of heart failure or arrythmia plus other non-cardiac clinical manifestations of *TTR*-related amyloidosis whose *TTR* gene was Sanger sequenced.

Sex	BCT	LEN	SR	SS	CD	ED	G	AVB	N/T	NP	MW	SN	OH	TOTAL
F	X	X	X	X	X									**5**
F	X	X	X	X										**4**
F	X		X		X									**3**
F	X	X	X	X	X		X			X				**7**
F	X	X	X	X	X									**5**
F	X	X	X	X							X		X	**6**
F	X	X	X	X		X								**5**
F	X	X	X	X		X								**5**
M	X	X	X	X				X				X		**6**
M	X	X	X											**3**
M	X	X	X	X				X	X		X			**7**
F	X	X	X			X								**4**
F	X	X	X	X										**4**
F	X	X		X			X							**4**
F	X	X	X	X						X	X	X		**7**
F	X	X	X	X	X	X			X	X				**8**
F	X	X		X	X	X	X							**6**
F	X	X		X			X	X						**5**
F	X	X	X	X										**4**
F	X	X		X		X	X		X					**6**
F	X	X	X	X										**4**
F	X	X	X	X	X		X							**6**
M	X		X											**2**
M	X	X	X			X								**4**
M	X	X	X											**3**
M	X	X	X	X					X					**5**
M	X	X	X	X										**4**
**TOTAL**	**27**	**25**	**23**	**21**	**7**	**7**	**6**	**3**	**4**	**3**	**3**	**2**	**1**	

BCT- Bilateral Carpal Tunnel
LEN- Lower Extremities Numbness
SR- Spinal Radiculopathy
SS- Spinal Stenosis
CD- Chronic Diarrhea
ED- Eye Diseases
G- Gastroparesis
AVB- AV Bundle Branch Block
N/T- Numbness or Tingling
NP- Neuropathic Pain

MW- Muscle Weakness
SN- Sensorimotor Neuropathy
OH- Orthostatic Hypotension

Table S3. Variants found by Sanger sequencing of patients of African American ancestry with a diagnosis of heart failure or arrythmia plus other non-cardiac clinical manifestations of *TTR*-related amyloidosis.

Bases	CHR POSITION	INTRON/ EXON/UTR	CLINVAR
G and A	31,592,439	INTRON	N/A
T and A	31,592,770	INTRON	N/A
C and G<br>A and C<br>T and C	31,598,550<br>31,598,696<br>31,599,077	INTRON<br>3′ UTR<br>INTRON	Benign (ClinVar)<br>Benign (ClinVar)<br>N/A
A and G	31,592,764	INTRON	N/A
T and C	31,599,077	INTRON	N/A
T and C	31,599,077	INTRON	N/A
T and C	31,599,077	INTRON	N/A
T and C	31,595,554	INTRON	N/A
T and C	31,599,077	INTRON	N/A
A and –	31,591,704	INTRON	N/A
T and A<br>G and A	31,592,770<br>31,592,764	INTRON<br>INTRON	N/A<br>N/A
A and G<br>T and C	31,599,020<br>31,599,077	INTRON<br>INTRON	N/A<br>N/A
T and C	31,599,077	INTRON	N/A
C and A	31,598,416	INTRON	N/A
T and C	31,599,077	INTRON	N/A
T and C	31,599,077	INTRON	N/A
T and C	31,599,077	INTRON	N/A
A and G	31,592,764	INTRON	N/A
T and C	31,599,077	INTRON	N/A
A and T	31,595,044	INTRON	N/A
A and T<br>T and G	31,595,044<br>31,594,941	INTRON<br>INTRON	N/A<br>N/A
A and G	31,592,764	INTRON	N/A
T and C	31,599,077	INTRON	N/A
T and C	31,595,088	INTRON	Likely benign (ClinVar)
A and G	31,595,088	INTRON	Likely benign (ClinVar)
A and G	31,592,764	INTRON	N/A
T and C	31,599,077	INTRON	N/A
A and G	31,598,733	3′ UTR	Not classified
T and C	31,598,733	3′ UTR	Not Classified
T and A	31,592,770	INTRON	N/A
A and C<br>T and C	31,598,696<br>31,599,077	3′ UTR<br>INTRON	Benign (ClinVar)<br>N/A
A and G	31,592,764	INTRON	N/A
T and C	31,599,077	INTRON	N/A
T and C	31,599,077	INTRON	N/A
T and C	31,599,077	INTRON	N/A
G and A	31,592,074	INTRON	N/A
C and T	31,592,074	INTRON	N/A
A and G	31,592,764	INTRON	N/A

T and C<br>T and G	31,595,088<br>31,595,335	INTRON<br>INTRON	N/A<br>N/A
T and C	31,599,077	INTRON	N/A
A and G	31,592,764	INTRON	N/A
A and G	31,599,020	INTRON	N/A
A and G	31,592,764	INTRON	N/A
T and C	31,595,088	INTRON	N/A
T and C	31,599,077	INTRON	N/A
A and G	31,595,088	INTRON	N/A
A and G	31,595,088	INTRON	N/A

Table S4. AA missense *TTR* variants in gnomAD (v4.0.0) by population.

**Chromosome**	18	18	18	18	18
**Position**	31598655	31595181	31595158	31592974	31598608
**rsIDs**	rs76992529	rs121918085	rs1254341785	rs28933979	rs1456101911
**Reference**	G	A	C	G	C
**Alternate**	A	T	T	A	A
**Protein Consequence**	p.Val142Ile	p.Ile88Leu	p.Thr80Ile	p.Val50Met	p.Thr126Asn
**Transcript Consequence**	c.424G>A	c.262A>T	c.239C>T	c.148G>A	c.377C>A
**VEP Annotation**	missense_variant	missense_variant	missense_variant	missense_variant	missense_variant
**ClinVar Clinical Significance**	Pathogenic/ Likely pathogenic	Pathogenic/ Likely pathogenic	Pathogenic/ Likely pathogenic	Pathogenic	Likely pathogenic
**Allele Count**	1433	21	4	92	1
**Allele Number**	1614082	1613992	780904	1614098	152230
**Allele Frequency**	0.000887811	1.30112E-05	5.12227E-06	5.69978E-05	6.56901E-06
**Homozygote Count**	8	0	0	0	0
**Hemizygote Count**	0	0	0	0	0
**Allele Count African/African American**	1273	4	1	1	1
**Allele Number African/African American**	75018	74924	59116	75034	41466
**Homozygote Count African/African American**	8	0	0	0	0
**Hemizygote Count African/African American**	0	0	0	0	0
**Allele Count Admixed American**	55	0	0	5	0
**Allele Number Admixed American**	60022	60000	59018	60016	15288

**Homozygote Count Admixed American**	0	0	0	0	0
**Hemizygote Count Admixed American**	0	0	0	0	0
**Allele Count Ashkenazi Jewish**	0	0	0	0	0
**Allele Number Ashkenazi Jewish**	29608	29604	24452	29604	3472
**Homozygote Count Ashkenazi Jewish**	0	0	0	0	0
**Hemizygote Count Ashkenazi Jewish**	0	0	0	0	0
**Allele Count East Asian**	1	0	0	2	0
**Allele Number East Asian**	44868	44884	41250	44886	5194
**Homozygote Count East Asian**	0	0	0	0	0
**Hemizygote Count East Asian**	0	0	0	0	0
**Allele Count European (Finnish)**	0	0	0	4	0
**Allele Number European (Finnish)**	63988	64030	63748	64014	10622
**Homozygote Count European (Finnish)**	0	0	0	0	0
**Hemizygote Count**	0	0	0	0	0

**European (Finnish)**					
**Allele Count Middle Eastern**	4	0	0	1	0
**Allele Number Middle Eastern**	6062	6084	4464	6062	316
**Homozygote Count Middle Eastern**	0	0	0	0	0
**Hemizygote Count Middle Eastern**	0	0	0	0	0
**Allele Count European (non-Finnish)**	30	17	0	62	0
**Allele Number European (non-Finnish)**	1180020	1179982	418132	1179988	68038
**Homozygote Count European (non-Finnish)**	0	0	0	0	0
**Hemizygote Count European (non-Finnish)**	0	0	0	0	0
**Allele Count Amish**	0	0	0	0	0
**Allele Number Amish**	910	912	912	912	912
**Homozygote Count Amish**	0	0	0	0	0
**Hemizygote Count Amish**	0	0	0	0	0
**Allele Count South Asian**	13	0	3	3	0
**Allele Number South Asian**	91078	91086	74622	91078	4836

**Homozygote Count South Asian**	0	0	0	0	0
**Hemizygote Count South Asian**	0	0	0	0	0
**Allele Count Remaining**	57	0	0	14	0
**Allele Number Remaining**	62508	62486	35190	62504	2086
**Homozygote Count Remaining**	0	0	0	0	0
**Hemizygote Count Remaining**	0	0	0	0	0

Method S1. TTR p.Val142Ile, rs76992529 Custom Taqman Genotyping Assay using probe fluorophores VIV and FAM to distinguish G from A.

TTR p.Val142Ile rs76992529 Seq:
CTACTCCTATTCCACCACGGCTGTC**[G/A]**TCACCAATCCCAAGGAATGAGGGAC

Primer locations:

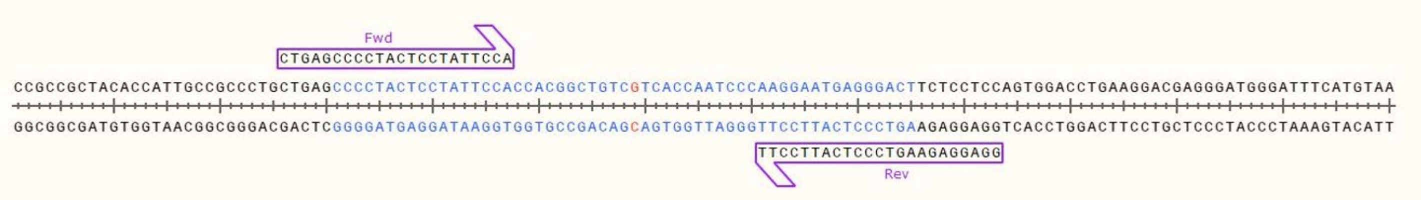

Method S2. TTR gene Sanger Sequencing.
Long PCR was performed to amplify the entire TTR gene encompassing 4 exons resulting in a 7792 bp product. The fragments were then gel isolated and Sanger sequenced (Genewiz) using sequencing primers at the 5' and 3' ends of all 4 exons.

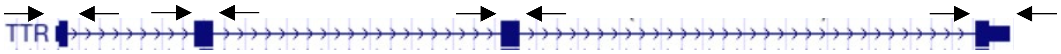

Location of Sanger Sequencing primers.

Agarose Gel Electrophoresis
Visual quality check on 7792 bp band:

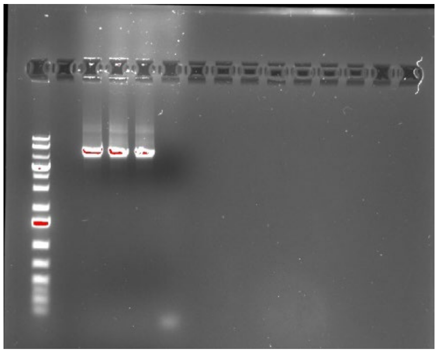

Amplified PCR product subject to electrophoresis at 110V for 28 minutes illustrating 3 *TTR* gene long PCR product bands.
